# Single-cluster pull-down assay for visualization and characterization of nanoclusters and condensates

**DOI:** 10.1016/j.xpro.2026.104597

**Published:** 2026-05-22

**Authors:** Tapas Paul, Sua Myong

**Affiliations:** 1 Program in Cellular and Molecular Medicine, Boston Children’s Hospital, Harvard Medical School, Boston, MA, USA

**Keywords:** Biophysics, Single-molecule Assays, Molecular Biology, Protein Biochemistry

## Abstract

Biomolecular condensates regulate cellular organization, but early assembly intermediates remain difficult to resolve. Here, we present single-cluster pull-down (SiCluP), a fluorescence microscopy assay to selectively immobilize nanoclusters under defined experimental conditions onto antibody-functionalized PEG surfaces for TIRF imaging and quantitative single-cluster analysis. We describe steps for surface preparation, nanocluster assembly within the flow chamber, selective immobilization, and time-resolved measurements. Using FUS as an example, SiCluP enables the quantification of assembly kinetics, relative cluster size, and molecular exchange at the single-cluster level.

For complete details on the use and execution of this protocol, please refer to Ge et al.^1^

## Highlights

• Instructions for preparing PEG-passivated antibody-functionalized flow chambers
• Steps for assembling and immobilizing nanoclusters in the SiCluP assay
• Guidance on acquiring real-time single-cluster imaging and kinetic measurements
• Procedures for analyzing cluster size, mixing, exchange, and FRAP dynamics

## Abstract (editor highlights)

Publisher’s note: Undertaking any experimental protocol requires adherence to local institutional guidelines for laboratory safety and ethics.

## Graphical abstract

**Figure:**
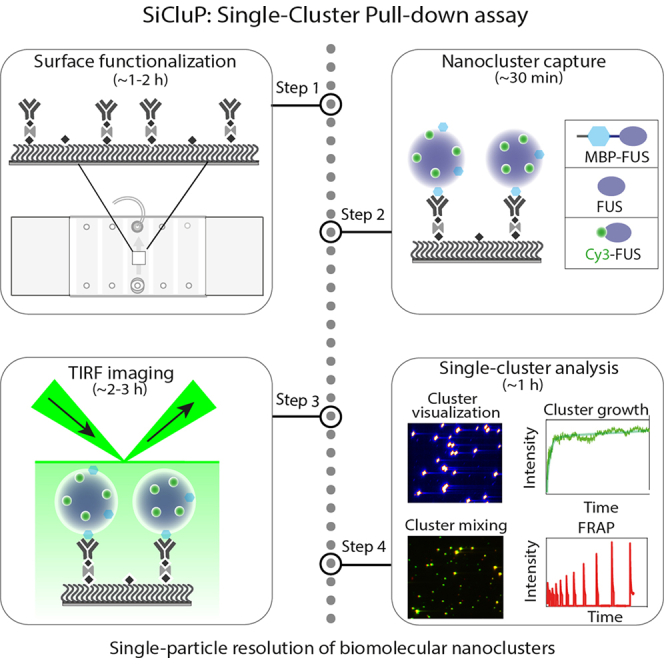

## Before you begin

### Overview

This protocol describes Single-Cluster Pull-down (SiCluP), a fluorescence microscopy-based assay that enables selective immobilization and quantitative analysis of biomolecular nanoclusters at the single-cluster level. In this approach, a small fraction of the protein of interest is affinity-tagged to enable antibody-mediated surface capture, while a fluorescently labeled fraction is included for visualization. Nanoclusters are assembled under defined conditions and monitored within the flow chamber during imaging, allowing real-time observation of cluster formation and growth. These nanoclusters are selectively immobilized on antibody-functionalized PEG-passivated surfaces for total internal reflection fluorescence (TIRF) imaging. SiCluP enables single-cluster resolution of assembly kinetics, relative cluster size, and molecular exchange dynamics.

### Innovation

Conventional bulk assays and microscopy approaches often lack the sensitivity to resolve early-stage biomolecular nanoclusters or quantify their dynamic properties at the single-cluster level. SiCluP integrates antibody-mediated surface capture with prism-type TIRF microscopy to enable selective immobilization and direct visualization of individual nanoclusters under defined solution conditions. Compared with conventional condensate assays, this protocol allows quantitative measurement of nanocluster assembly kinetics, relative cluster size, inter-cluster mixing, molecular exchange, and fluorescence recovery from individual assemblies. The method also combines customizable affinity-tag capture strategies with standard single-molecule imaging workflows, allowing adaptation to diverse protein and nucleic acid-associated condensate systems. In addition, the accompanying analysis pipeline enables quantitative extraction of kinetic and structural parameters from single-cluster fluorescence trajectories.

### Experimental planning and setup

**Timing: ∼1 h (excluding protein purification and slide passivation)**

1. Prepare protein variants required for SiCluP.
  a. Purify the protein of interest in both untagged and tagged forms suitable for antibody-based capture.
  b. Prepare a fluorescently labeled fraction of untagged protein for single-cluster visualization.
2. Minimize pre-aggregation and variability.
  a. Clarify protein stocks by high-speed centrifugation prior to use.
  b. Avoid repeated freeze-thaw cycles.
  c. Maintain consistent buffer composition, temperature, and incubation times during cluster assembly.
3. Prepare PEG-passivated slides and flow chambers.
  a. Use PEGylated slides compatible with antibody functionalization and low nonspecific binding.
  b. Prepare slides in advance if desired and store appropriately until use.
4. Calibrate the TIRF microscope.
  a. Verify laser alignment, focus, and channel registration using multicolor fluorescent bead standards prior to imaging.
  b. Follow institutional laser safety guidelines.
5. Prepare fresh imaging buffer.
  a. Prepare imaging buffer immediately before experiments.
  b. Ensure oxygen scavenging reagents and antifade components are active.

### Purified protein preparation

**Timing: ∼1–2 days or variable (depending on expression system)**

6. Obtain purified protein capable of forming nanoclusters or condensates.
  a. Purify the protein of interest using workflows appropriate for the protein system.
  b. For FUS, purify untagged and MBP-tagged constructs.^1^^,^^2^
  c. Prepare other cluster-forming proteins using suitable recombinant expression and purification strategies.
7. Prepare both tagged and untagged protein fractions.
  a. Prepare a majority untagged protein population to preserve native clustering behavior.
  b. Prepare a small fraction of tagged protein for surface capture.
  c. Prepare a fluorescently labeled fraction, typically derived from the untagged construct, for visualization.

***Note:*** In this study, tagged protein and fluorescently labeled untagged protein concentrations were kept constant while unlabeled untagged protein concentration was varied. Typical concentrations were ∼30–50 nM tagged protein and ∼5–10 nM labeled untagged protein.

8. Fluorescent labeling.
  a. Label purified untagged protein using an appropriate dye-conjugation strategy (e.g., maleimide or NHS chemistry).
  b. Perform fluorophore labeling (e.g., Cy3 or Cy5 NHS ester) using standard protocols.^1^
  c. Quantify labeling efficiency and keep labeling low (e.g., ≤10% of the protein population).
  d. Remove excess free dye prior to use.
  e. Verify that labeling does not significantly perturb clustering behavior.
9. Quality control.
  a. Confirm protein purity (>90%) by SDS-PAGE.
  b. Clarify protein stocks by high-speed centrifugation (e.g., 15,000–20,000 × g for 10–15 min at 4 °C) prior to cluster assembly.
10. Storage and handling.
  a. Aliquot purified protein to avoid repeated freeze-thaw cycles.
  b. Store under conditions that minimize spontaneous aggregation.
  c. Thaw samples on ice and use immediately.

### Preparation of PEG-passivated slides

**Timing: ∼1–2 days**

PEG-passivated slides are required to minimize nonspecific adsorption and enable antibody-based surface functionalization.^3^ The procedure below outlines glass cleaning, silanization, and PEG coupling.

11. Cleaning and surface activation
  a. Place glass slides (3″ × 1″, ∼1 mm thick) and coverslips (e.g., 24 × 40 mm) in a glass staining jar.
  b. If reusing slides, soak in ethanol or acetone for several hours to remove residual adhesives and contaminants. Rinse thoroughly with water before proceeding.
  c. Submerge slides and coverslips in 5% (w/v) laboratory detergent solution and sonicate for 5–10 min at 20–25°C.
  d. Rinse extensively with running water followed by Milli-Q water.
  e. Treat slides and coverslips with 1 M KOH and sonicate for 30–45 min to activate the glass surface.
  f. Discard KOH and rinse repeatedly with Milli-Q water until completely neutralized.
  g. Dry slides and coverslips under a nitrogen stream.
  h. Flame-dry for 1 to 2 s each surface using a propane torch to remove residual moisture and organic contaminants. Allow surfaces to cool before proceeding.
12. Amino-silanization
  a. Prepare a silanization solution consisting of methanol containing acetic acid and aminosilane (e.g., N-(2-aminoethyl)-3-aminopropyltrimethoxysilane) (e.g., 95% methanol, 5% acetic acid, and ∼1–2% aminosilane, v/v).
  b. Fully immerse slides and coverslips in the solution using a slide holder or glass staining jar.
  c. Incubate in the dark for ∼30–40 min without agitation.
  d. Rinse thoroughly with methanol followed by Milli-Q water.
  e. Dry completely under nitrogen.

**CRITICAL:** Proper drying at this stage is essential to prevent premature hydrolysis of silane groups.

13. PEG coupling.
  a. Prepare freshly made 100 mM sodium bicarbonate buffer (pH ∼8.3) by dissolving 84 mg sodium bicarbonate in 10 mL Milli-Q water.
  b. Dissolve mPEG-SVA together with biotin-PEG-SVA (typically ∼1-2% molar ratio) in freshly prepared 100 mM sodium bicarbonate buffer (e.g., total PEG ∼100 mg in ∼1 mL buffer, with 1–2 mg biotin-PEG-SVA and the remainder mPEG-SVA). Mix gently and centrifuge (e.g., 5,000–10,000 × *g* for 1–2 min at 20–25°C) to remove particulates.
  c. Immediately apply ∼60–80 μL of PEG solution to each slide and overlay with a coverslip to form a uniform thin film. Avoid introducing air bubbles.
  d. Incubate slides in a humidified chamber in the dark for 3–5 h at 20–25°C.

***Note:*** For experiments requiring extremely low nonspecific background, PEGylation may be repeated by reapplying freshly prepared PEG solution after separating the slide-coverslip pair, washing, and drying the surfaces.

14. Washing and storage.
  a. After incubation of PEGylation, carefully separate the slide and coverslip using forceps and rinse thoroughly with Milli-Q water.
  b. Dry under nitrogen.
  c. Store dried PEG-passivated slides in sealed containers at −20 °C until use. Mark the non-functionalized side of the slide or coverslip (e.g., with a diamond pen or marker) during preparation to enable identification of the PEG-coated surface during assembly.

***Note:*** Slides can typically be stored for several weeks without significant loss of passivation quality.

## Key resources table

**Table:** 

REAGENT or RESOURCE	SOURCE	IDENTIFIER
**Antibodies**		

Biotin-conjugated anti-MBP	Rockland	Cat#200406385S; used at 5–10 nM

**Chemicals, peptides, and recombinant proteins**		

Alconox powdered detergent	Fisher Scientific	Cat#16000104
Biotin-PEG-SVA (MW 5,000)	Laysan Bio Inc.	Cat#Biotin-PEG-SVA-5000-100mg
Cy3 NHS Ester	Cytiva	CAT#PA13101
Cy5 NHS Ester	Cytiva	CAT#PA15100
Catalase (bovine liver)	Sigma-Aldrich	Cat#C3155
Glucose oxidase (Aspergillus niger)	Sigma-Aldrich	Cat#G2133
mPEG-SVA (MW 5,000)	Laysan Bio Inc.	Cat#MPEG-SVA-5000-1g
N-(2-aminoethyl)-3-aminopropyltrimethoxysilane	United Chemical Technologies	Cat#1760-24-3
NeutrAvidin protein	Thermo Scientific	Cat#31000
Trolox	Fisher Scientific	Cat#AC218940010
Recombinant FUS protein	This study	N/A

**Software and algorithms**		

IDL	Harris Geospatial	https://www.l3harrisgeospatial.com/Software-Technology/IDL
MATLAB	Mathworks	https://www.mathworks.com/products/matlab.html
smCamera	Available at Roy et al.^4^	https://cplc.illinois.edu/research/tools

**Other**		

Rapid-curing epoxy adhesive	All-Spec	Cat#14250
Fusion 200 Touch syringe pump	Fisher Scientific	Cat#NC0670590
PTFE tubing (Size 26)	Weico Wire	Cat#ETT-26
Quartz microscope slides	G Finkenbeiner Inc.	N/A
Double-sided tape (665)	Office Depot	Cat#391775
TetraSpeck microspheres (0.1 μm)	Thermo Fisher	Cat#T7279
TIRF microscope	Olympus	N/A
532-nm and 634-nm diode lasers	Compass 315M; Coherent, Santa Clara, CA	N/A
0.22-μm membrane	Sigma-Aldrich	Cat#SLMP025SS

## Materials and equipment

A prism-type total internal reflection fluorescence (TIRF) microscope equipped with 532-nm and 634-nm solid-state lasers is required for single-cluster imaging.^4^^,^^5^ A syringe pump is required for real-time flow experiments.

**Table:** 10× T50 Buffer (10 mL)

Reagent	Final concentration (mM)	Amount
1 M Tris, pH 7.4	100 mM	1 mL
5 M NaCl	500 mM	1 mL
Milli-Q H_2_O	N/A	8 mL
**Total**	**N/A**	**10 mL**

**Storage:** Store at 20–25°C for up to 6 months.

**Table:** 10× TN100 Buffer (10 mL), Use at 1× as experimental buffer

Reagent	Final concentration (mM)	Amount
1 M Tris, pH 7.4	100 mM	1 mL
5 M NaCl	1000 mM	2 mL
Milli-Q H_2_O	N/A	7 mL
**Total**	**N/A**	**10 mL**

**Storage:** Store at 20–25°C for up to 6 months.

**Table:** 100X Glucose oxidase-catalase (Glu-oxy) stock (100 μL)

Reagent	Final concentration (mM or μM)	Amount
Glucose Oxidase	N/A	10 mg
Catalase	N/A	2 μL of (4μg/mL)
T50 Buffer	N/A	98 μL
**Total**	**N/A**	**100** μ**L**

**Storage:** Store at 4 °C for up to 8 weeks.

**Table:** Imaging buffer (prepare fresh) (100 μL)

Reagent	Final concentration (mM or μM)	Amount
10× experimental buffer	1× buffer	10 μL
20% (w/v) glucose	0.4% (w/v)	1 μL
100× Glu-oxy	1×	1 μL
10 mM Trolox	∼10 mM	88 μL
**Total**	**N/A**	**100 μL**

**Storage:** Prepare fresh immediately before use; do not store.

All 10× stock buffers should be filtered through a 0.22-μm membrane prior to use. Prepare the imaging buffer immediately prior to each experiment. The glucose oxidase-catalase (Glu-oxy) stock may be stored at 4 °C for up to 8 weeks. A 10 mM Trolox stock solution can be stored at −20 °C for up to 6 months or at 4 °C for up to 8 weeks.

***Alternatives:*** Protocatechuic acid-protocatechuate-3,4-dioxygenase (PCA-PCD) can be used instead of glucose oxidase and glucose.

## Step-by-step method details

### Flow chamber assembly

**Timing: ∼1 h**

**Figure 1:**
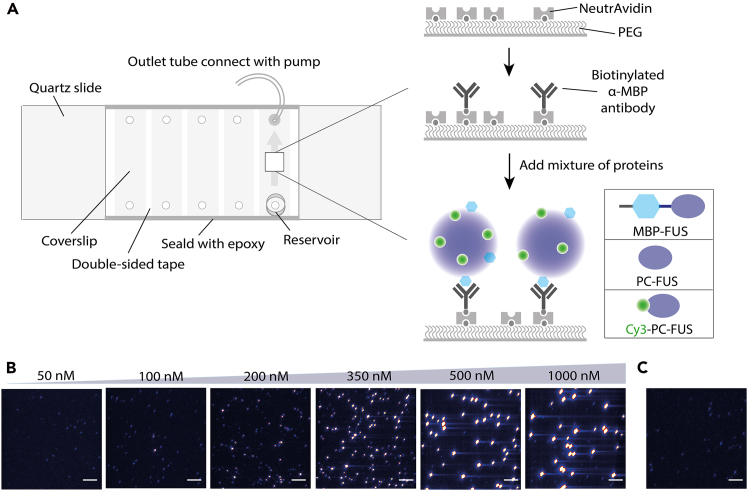
Single-Cluster Pull-down (SiCluP) assay and FUS nanocluster capture (A) Schematic of the microfluidic flow chamber used for SiCluP. The chamber contains a buffer reservoir at one end and an outlet connected to a syringe pump for controlled flow. Biotinylated anti-MBP antibody is immobilized on PEG-passivated surfaces via biotin–NeutrAvidin interactions. FUS nanoclusters containing a small fraction of MBP-FUS are selectively captured through antigen–antibody binding. (B) Representative TIRF images of surface-captured FUS nanoclusters at increasing total FUS concentrations (50 nM, 100 nM, and ≥200 nM). At lower concentrations, signals are diffraction-limited and dim. At higher concentrations, brighter and larger clusters are observed. Scale bars, 5 μm in all fluorescence images. (C) Representative TIRF image of 500 nM FUS containing the same fraction of labeled FUS but in the absence of MBP-FUS. No clusters were observed, indicating minimal nonspecific surface binding.

Here, we describe steps for assembling PEG-passivated slides into microfluidic chambers for SiCluP experiments (Figure 1A).

1. Fabrication of the flow channel.
  a. Cut two parallel strips of double-sided tape (∼1 mm wide) and place them lengthwise on a PEG-passivated slide to define the flow channel boundaries (Figure 1A).
  b. Position the tape so that the channel spans the pre-drilled inlet and outlet holes (drilled prior to PEG passivation).
  c. Place the PEG-passivated coverslip with the PEG-coated (functionalized) side facing inward on top of the tape strips to form the channel.
  d. Apply gentle pressure along the tape edges (e.g., using a pipette tip) to ensure proper sealing.
  e. Seal exposed edges using rapid-curing epoxy and allow curing for 5–10 min.
  f. Attach inlet reservoirs and outlet tubing using trimmed pipette tips and epoxy if flow experiments are required.
  g. Slowly fill the channel with buffer to verify that no leakage or trapped air bubbles are present.
  h. The flow chamber is now ready for surface functionalization.

### Instrument calibration

**Timing: ∼30 min**

Here, we describe calibration of the TIRF microscope for channel registration and pixel-size determination.

2. Preparation of bead slide
  a. Dilute 1 μL TetraSpeck fluorescent beads in 99 μL T50 buffer and vortex for 2–3 s to ensure uniform dispersion.
  b. Apply 50 μL diluted bead solution to a non-PEGylated chamber (assembled using double-sided tape spacers without reservoir or outlet tubing).
  c. Incubate 3–5 min, wash gently with ∼100 μL T50 buffer to remove unbound beads, and seal the chamber using epoxy at the edges.
  d. Mount the bead slide on the TIRF microscope and adjust the focus to the surface plane. Optimize laser alignment and illumination to obtain sharp, diffraction-limited bead signals in all channels.
  e. Record short movies (∼2 s) under identical imaging conditions (laser power, exposure time, and camera gain) to generate channel mapping files.

***Note:*** Optimal bead density is approximately 300–400 spots per 2,500 μm^2^ imaging area. Pixel size calibration may be performed using bead standards of defined diameter.

### Surface functionalization

**Timing: ∼20 min**

Here, we describe functionalization of PEG-passivated chambers for selective nanocluster capture (Figure 1).

3. Flow chamber preparation.
  a. Introduce ∼100 μL NeutrAvidin (100 μg/mL in experimental buffer) into the chamber via the reservoir or by pipetting into the inlet, and incubate for 1-2 min. NeutrAvidin solution can be prepared fresh or thawed from aliquots stored at −20 °C.
  b. Wash with ∼100–200 μL experimental buffer.
  c. Introduce ∼100 μL biotin-conjugated anti-MBP antibody (5–10 nM in experimental buffer).
  d. Incubate for 1 to 2 min and wash thoroughly with ∼100–200 μL experimental buffer.
  e. The chamber is now ready for cluster pull-down.

**CRITICAL:** Optimize antibody concentration to achieve sparse cluster density (∼100–150 clusters per field of view). Excess antibody increases nonspecific capture and cluster overlap.

### Nanocluster assembly and loading

**Timing: ∼20 min**

**Figure 2:**
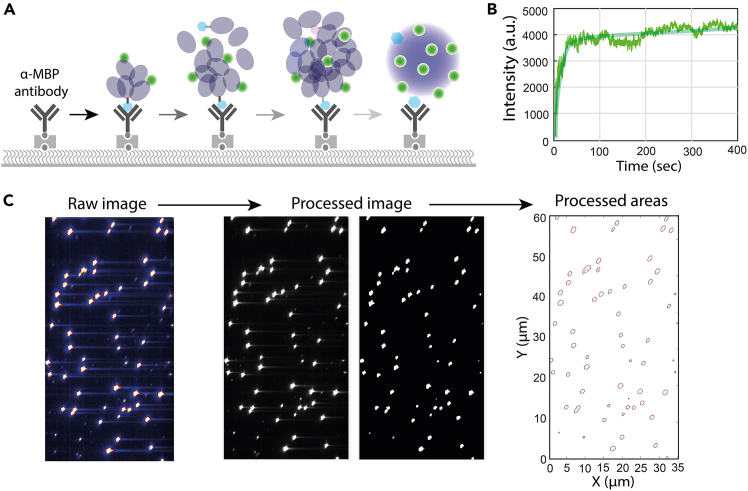
FUS nanocluster assembly and size analysis (A) Schematic of FUS nanocluster assembly and growth within the flow chamber over time. (B) Representative single-cluster fluorescence intensity time traces showing nanocluster growth over time. (C) TIRF field-of-view image of captured FUS nanoclusters and corresponding processed image used for relative cluster size estimation based on calibrated pixel size.

Here, we describe preparation and loading of nanocluster mixtures for in situ assembly within the flow chamber (Figure 2).

4. Make nanocluster mixture.
  a. Prepare a mixture containing MBP-FUS (∼30–50 nM), fluorophore-labeled FUS (∼5–10 nM), and variable concentrations of unlabeled FUS (∼50–500 nM) in experimental buffer.
  b. Mix the components gently and immediately load the mixture into the reservoir, where nanocluster assembly occurs within the chamber over time.

**CRITICAL:** The tagged protein concentration should be kept low to minimize perturbation of native assembly behavior while ensuring efficient surface capture.

***Note:*** In this assay, a fixed low concentration of tagged protein was used while total protein concentration was varied.

***Note:*** Optimal protein concentrations may vary depending on the protein system and buffer conditions and should be empirically optimized.

### Single-cluster capture and visualization

**Timing: ∼1–3 h**

Here, we describe selective capture of nanoclusters on functionalized PEG surfaces and their visualization by TIRF microscopy (Figure 1).

5. Cluster capture and visualization.
  a. Place the functionalized chamber on the TIRF microscope.
  b. Introduce ∼100 μL of the nanocluster mixture into the chamber via the reservoir or inlet.
  c. Allow clusters to bind for 1 to 2 min under static conditions (no-flow) with the outlet pump stopped.
  d. Proceed directly to imaging without washing to avoid perturbation or loss of weakly bound clusters.
  e. Record short movies (∼2 s) at multiple imaging areas using appropriate TIRF settings (e.g., 532-nm or 634-nm excitation, ∼50–100 ms exposure time per frame, and constant low laser power to minimize photobleaching).

***Note:*** Evaluate nonspecific binding using samples lacking tagged protein (i.e., containing fluorescently labeled untagged protein only). For FUS, minimal surface binding was observed in the absence of tagged protein, indicating that immobilization is primarily mediated by antibody–tag interactions (Figure 1C).

### Real-time nanocluster assembly kinetics

**Timing: ∼1–3 h**

Here, we describe real-time imaging of nanocluster assembly to quantify cluster growth kinetics (Figure 2).

6. Assembly kinetics acquisition.
  a. Pre-mix tagged protein (∼30–50 nM) and fluorescently labeled untagged protein (∼5–10 nM) in experimental buffer at 20–25°C.
  b. Initiate assembly by adding unlabeled protein at the desired concentration (e.g., 200–1000 nM) and mix gently.
  c. Immediately load ∼100 μL of the mixture prepared in steps 6a and 6b into the reservoir.
  d. Begin controlled flow (∼0.1 mL/min) using a syringe pump while recording continuously.
  e. Record continuous movies at defined time resolution (e.g., 100 ms/frame) for an appropriate duration (e.g., 5–10 min for FUS) to capture cluster growth dynamics.

***Note:*** Fluorescence intensity increases over time as clusters grow.

**CRITICAL:** Maintain constant laser power, camera settings, and ambient temperature throughout kinetic acquisition. Experiments were performed at 20–25°C; fluctuations in temperature may affect cluster assembly kinetics.

### Cluster size determination

**Timing: ∼1–3 h**

Here, we describe acquisition and analysis of fluorescence images for relative nanocluster size determination.

7. Size measurements.
  a. Acquire short movies (∼2 s each) at defined time intervals under identical imaging conditions. For each condition, record movies from multiple fields of view (e.g., 5–10 movies).
  b. Process images using a custom MATLAB script (see Data and Code Availability) to segment individual clusters and determine their projected area in pixels.
    ***Note:*** Apply background subtraction and intensity thresholding consistently across datasets. If these steps are performed manually, use identical parameters for all conditions.
  c. Convert the measured cluster area (in pixels) to physical units using the calibrated pixel size (μm/pixel). Assuming approximately circular clusters, calculate the effective diameter from the measured area (Figure 2C).
    **CRITICAL:** Avoid detector saturation during image acquisition, as saturated signals can distort apparent cluster area measurements. Adjust laser power and exposure time to keep signals within the camera’s dynamic range.
    ***Note:*** TIRF-based measurements provide relative projected size estimation. Absolute size accuracy depends on proper pixel calibration and segmentation parameters.

### Cluster mixing and exchange

**Timing: ∼1–3 h**

**Figure 3:**
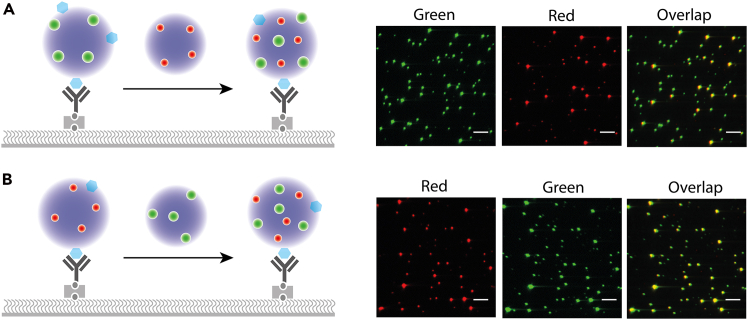
Nanocluster mixing experiments (A, B) Dual-color mixing of FUS nanoclusters. Cy3-labeled clusters (A) or Cy5-labeled clusters (B) were immobilized on the surface, followed by introduction of the complementary labeled nanocluster population. Colocalization of green and red fluorescence indicates nanocluster mixing. Scale bars, 5 μm in all fluorescence images.

Here, we describe dual-color imaging experiments to assess inter-cluster mixing dynamics (Figure 3).

8. Cluster mixing (two-color colocalization).
  a. Immobilize Cy3-labeled clusters on the surface as described in Section 5. Fluorescent labeling (e.g., Cy3 or Cy5) was performed using standard dye-conjugation chemistry (e.g., NHS or maleimide), and labeling efficiency was kept low (≤10%) to minimize perturbation of clustering behavior.
  b. Introduce ∼100 μL Cy5-labeled clusters into the chamber via controlled flow (∼0.05–0.1 mL/min) using a syringe pump.
  c. Acquire dual-color images at identical positions over time (e.g., every 5–10 s for ∼5–10 min for FUS) to monitor colocalization.
  d. Repeat with reversed labeling order to confirm reproducibility.

***Note:*** Avoid air bubbles during flow to prevent disturbance of immobilized clusters.

***Note:*** For FUS, colocalization increases over time and approaches a steady level, indicating progressive inter-cluster mixing rather than instantaneous equilibration.

### Molecular exchange measurements

**Timing: ∼1–3 h**

Here, we describe measurements of molecular exchange between solution-phase proteins and immobilized nanoclusters, distinct from inter-cluster mixing described in Section 8.

9. Exchange with cluster
  a. Immobilize labeled nanoclusters on the surface as described in Section 5.
  b. Introduce differently labeled or unlabeled clusters via slow flow (∼0.05 mL/min) using a syringe pump.
  c. Record long movies (∼10 min) under constant imaging conditions (e.g., 50–100 ms/frame).
  d. Extract single-cluster fluorescence intensity traces over time and quantify changes in intensity to assess molecular exchange dynamics.

***Note:*** In contrast to Section 8 (cluster mixing), this assay monitors exchange of molecules between solution and pre-existing clusters. For FUS, gradual changes in fluorescence intensity over time indicate continuous molecular exchange.

### FRAP analysis of surface-tethered nanoclusters

**Timing: ∼1–3 h**

**Figure 4:**
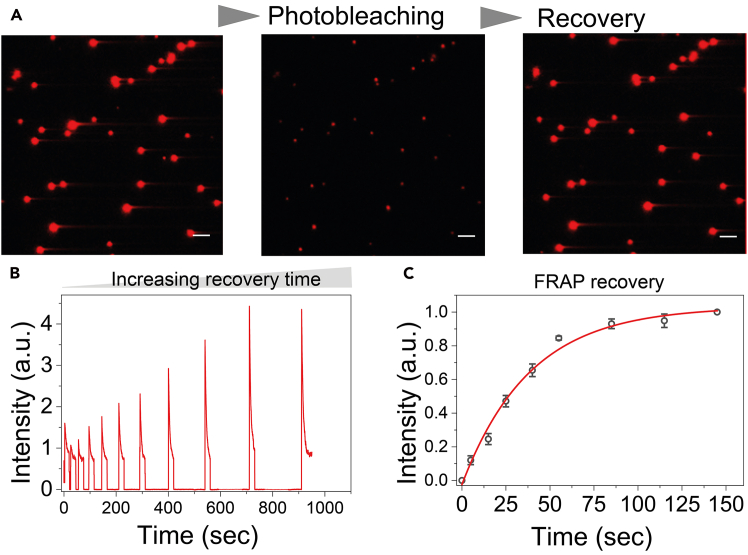
Fluorescence recovery after photobleaching (FRAP) of surface-tethered nanoclusters (A) Representative TIRF image of Cy5-labeled FUS nanoclusters subjected to periodic photobleaching and recovery. Scale bars, 5 μm in all fluorescence images. (B) Example single-cluster FRAP recovery traces. (C) Quantification of recovered fluorescence intensity over time, used to extract molecular exchange kinetics.

Here, we describe fluorescence recovery after photobleaching (FRAP) measurements to assess internal molecular mobility within nanoclusters (Figure 4).

10. FRAP measurements.
  a. Immobilize Cy5-labeled nanoclusters on the surface as described above.
  b. Fix the imaging region and avoid stage movement during the experiment.
  c. Photobleach selected region of clusters using high laser power (e.g., near-maximal laser intensity for ∼1–2 min) to achieve substantial fluorescence reduction.
  d. Record fluorescence recovery at defined recovery intervals (e.g., every 5–10 s over 5–180 s) under low laser power to minimize additional photobleaching.
  e. Extract single-cluster fluorescence intensity traces and fit recovery curves to single- or double-exponential models to obtain characteristic recovery times.

**CRITICAL:** Maintain constant laser power, exposure time, and camera gain across experiments. Avoid detector saturation and ensure consistent bleaching conditions.

***Note:*** Cy5 is recommended for FRAP due to more efficient photobleaching compared to Cy3. For FUS nanoclusters, partial fluorescence recovery is typically observed, indicating dynamic molecular exchange within clusters.

## Expected outcomes

Successful completion of this protocol enables visualization and quantitative analysis of individual nanoclusters immobilized on PEG-passivated surfaces. Under optimized antibody and protein concentration conditions, sparse surface densities (∼100–150 clusters per field of view) should be observed (Figure 1).

Single-cluster time traces allow quantification of early assembly kinetics and relative cluster growth (Figure 2). In dual-color experiments, co-localization analysis enables assessment of inter-cluster mixing and molecular exchange (Figure 3). FRAP measurements of surface-tethered nanoclusters reveal fluorescence recovery dynamics consistent with internal molecular mobility (Figure 4).

Although demonstrated here using FUS, the SiCluP assay can be adapted to other cluster-forming or phase-separating proteins by employing appropriate affinity tags and corresponding biotinylated capture reagents. For nucleic acid-associated assemblies, biotinylated DNA or RNA may be used as an alternative surface tethering strategy.

## Quantification and statistical analysis

Raw movies are processed using a calibrated channel-mapping file generated during instrument calibration. Single-cluster fluorescence time traces are extracted using IDL-based processing scripts (see Data and Code Availability), followed by downstream analysis in MATLAB. Image visualization and spot inspection may be performed using ImageJ or equivalent software.

### Cluster size analysis

Cluster size is determined from the projected cluster area obtained by image segmentation and converted to physical units using calibrated pixel size. This metric provides a relative estimate of cluster size across conditions.

### Assembly kinetics analysis

In this study, assembly kinetics were quantified using changes in cluster area over time. Fluorescence intensity may also be used as an alternative metric, particularly when segmentation is challenging or when clusters remain diffraction-limited. The choice of metric depends on image quality and cluster morphology.

### Molecular exchange analysis

Exchange kinetics are analyzed by extracting single-cluster fluorescence intensity traces over time. Changes in intensity reflect incorporation or loss of labeled molecules and can be used to assess exchange dynamics. When appropriate, intensity traces may be fitted to exponential models to estimate characteristic exchange times.

### Statistical analysis

Statistical comparisons between experimental conditions should be performed using appropriate tests based on data distribution. For pairwise comparisons, an unpaired two-tailed Student’s *t* test may be used when normality assumptions are satisfied. Multiple independent slide preparations and biological replicates are recommended to ensure reproducibility.

## Limitations

This protocol requires access to a prism-type TIRF microscope, which may limit accessibility. Surface passivation quality strongly influences nonspecific binding; optimization may be necessary for highly adhesive or aggregation-prone proteins.

Efficient cluster capture requires careful optimization of antibody concentration and tagged protein fraction to achieve sparse surface densities (∼100–150 clusters per field of view). The assay typically requires three protein variants: tagged, untagged, and fluorescently labeled constructs.

Fluorescence measurements are constrained by camera dynamic range. At high cluster density or brightness, signal saturation may limit quantitative analysis. Early assembly kinetics are therefore more reliably quantified than late-stage cluster growth.

Relative cluster size estimation depends on proper pixel calibration and consistent imaging parameters across experimental conditions. Complementary techniques such as nanoparticle tracking analysis (NTA) may be used to independently estimate cluster size and concentration.

## Troubleshooting

### Problem 1

Difficulty locating the TIRF illumination plane (step 2).

#### Possible cause

Improper prism coupling, incorrect incident angle, or misalignment of the microscope stage.

### Potential solution

Use a fluorescent bead calibration slide to identify the evanescent field region. Adjust the XYZ stage and prism alignment until bright, sharply defined bead signals are observed under TIRF illumination. Once the correct TIRF plane is established, replace with the experimental slide and fine-adjust focus using surface-associated features or weak background fluorescence.

### Problem 2

Too many or too few clusters immobilized on the surface (step 3 and step 5).

#### Possible cause

Suboptimal antibody concentration, incubation time, or tagged protein fraction.

### Potential solution

Optimize antibody concentration during surface functionalization. If cluster density is too high, increase antibody dilution or reduce incubation time. If density is too low, decrease dilution or increase incubation time. Adjust the tagged protein fraction if necessary. Aim for ∼100–150 clusters per field of view.

### Problem 3

Nonspecific binding of nanoclusters to the surface (step 3 and step 5).

#### Possible cause

Incomplete PEG passivation, insufficient washing, or highly adhesive protein assemblies.

### Potential solution

Include a control sample lacking tagged protein to assess nonspecific adsorption. If background persists, perform additional surface blocking using biotin (1 mM), BSA (0.4 mg/mL), and yeast tRNA (0.2 mg/mL) prior to sample introduction.^6^ Verify PEG passivation quality and consider repeating PEGylation.

### Problem 4

Fluorescence signal saturation during cluster growth measurements (step 6 and step 7).

#### Possible cause

Camera dynamic range exceeded by bright clusters or excessive laser power.

### Potential solution

Reduce laser intensity or exposure time to prevent detector saturation. Quantify early assembly kinetics before signal saturation occurs. Maintain consistent imaging parameters across conditions to ensure comparability.

### Problem 5

Inconsistent cluster size or FRAP measurements across experiments (step 7 and step 10).

#### Possible cause

Variation in laser power, exposure time, camera gain, or day-to-day instrument drift.

### Potential solution

Maintain constant imaging settings across experiments. Include a standard control condition for normalization when comparing experiments performed on different days. Verify pixel calibration prior to data acquisition.

### Problem 6

Disturbance of immobilized clusters during mixing experiments (step 8 and step 9).

#### Possible cause

Excessive flow rate or mechanical perturbation during buffer exchange.

### Potential solution

Introduce secondary clusters using controlled syringe pump flow at low flow rates appropriate for the chamber geometry. Avoid rapid buffer exchange and minimize direct pipetting into the chamber after initial immobilization.

### Problem 7

Inefficient photobleaching during FRAP experiments (step 10).

#### Possible cause

Fluorophore photostability, insufficient laser power, or rapid molecular exchange.

### Potential solution

Use fluorophores with higher photobleaching efficiency (e.g., Cy5). Optimize bleaching duration and laser intensity while minimizing photodamage. Ensure that pre-bleach baseline fluorescence is stable before initiating bleaching.

## Resource availability

### Lead contact

Further information and requests for resources and reagents should be directed to and will be fulfilled by the lead contact, Tapas Paul (tapas.paul@childrens.harvard.edu).

### Technical contact

Technical questions on executing this protocol should be directed to and will be answered by the technical contacts, Tapas Paul (tapas.paul@childrens.harvard.edu) and Sua Myong (sua.myong@childrens.harvard.edu).

### Materials availability

This study did not generate new unique reagents.

### Data and code availability

Single-molecule data acquisition and analysis packages can be obtained freely from the CPLC website (https://cplc.illinois.edu/research/tools). MATLAB code associated with this manuscript is archived on Zenodo and available at https://doi.org/10.5281/zenodo.19891953. IDL and MATLAB can be obtained with academic or individual licenses from their respective distributors. Any additional information required to reanalyze the data reported in this paper is available from the lead contact upon request.

## Acknowledgments

This project was supported by the grants from RF1 NS113636-01 and R01 AG071326-01.

## Author contributions

Conceptualization, T.P. and S.M.; methodology, T.P.; writing, T.P.; supervision, S.M.

## Declaration of interests

The authors declare no competing interests.
